# Conventional magnetic resonance imaging key features for distinguishing pathologically confirmed corticobasal degeneration from its mimics: a retrospective analysis of the J-VAC study

**DOI:** 10.1007/s00234-024-03432-w

**Published:** 2024-07-22

**Authors:** Keita Sakurai, Aya M. Tokumaru, Mari Yoshida, Yuko Saito, Koichi Wakabayashi, Takashi Komori, Masato Hasegawa, Takeshi Ikeuchi, Yuichi Hayashi, Takayoshi Shimohata, Shigeo Murayama, Yasushi Iwasaki, Toshiki Uchihara, Motoko Sakai, Ichiro Yabe, Satoshi Tanikawa, Hiroshi Takigawa, Tadashi Adachi, Ritsuko Hanajima, Harutoshi Fujimura, Kentaro Hayashi, Keizo Sugaya, Kazuko Hasegawa, Terunori Sano, Masaki Takao, Osamu Yokota, Tomoko Miki, Michio Kobayashi, Nobutaka Arai, Takuya Ohkubo, Takanori Yokota, Keiko Mori, Masumi Ito, Chiho Ishida, Jiro Idezuka, Yasuko Toyoshima, Masato Kanazawa, Masashi Aoki, Takafumi Hasegawa, Hirohisa Watanabe, Atsushi Hashizume, Hisayoshi Niwa, Keizo Yasui, Keita Ito, Yukihiko Washimi, Akatsuki Kubota, Tatsushi Toda, Kenji Nakashima, Ikuko Aiba, J-VAC study group

**Affiliations:** 1 Department of Radiology, National Center for Geriatrics and Gerontology, Obu, Aichi 474-8511 Japan; 2 Department of Diagnostic Radiology, Tokyo Metropolitan Institute for Geriatrics and Gerontology, 35-2 Sakae-Cho, Itabashi-Ku, Tokyo, 173-0015 Japan; 3 Department of Neuropathology, Institute for Medical Science of Aging, Aichi Medical University, Nagakute, Aichi 480-1195 Japan; 4 Department of Neuropathology (the Brain Bank for Aging Research), Tokyo Metropolitan Institute for Geriatrics and Gerontology, Itabashi, Tokyo 173-0015 Japan; 5 Department of Pathology and Laboratory Medicine, National Center of Neurology and Psychiatry, National Center Hospital, Kodaira, Tokyo, 187-8551 Japan; 6 Department of Neuropathology, Hirosaki University Graduate School of Medicine, Hirosaki, Aomori, 036-8562 Japan; 7 Department of Laboratory Medicine and Pathology (Neuropathology), Tokyo Metropolitan Neurological Hospital, Fuchu, Tokyo, 183-0042 Japan; 8 Department of Brain & Neurosciences, Tokyo Metropolitan Institute of Medical Science, Setagaya, Tokyo, 156-8506 Japan; 9 Department of Molecular Genetics, Brain Research Institute, Niigata University, Chuo, Niigata, 951-8585 Japan; 10 Department of Neurology, Gifu University Graduate School of Medicine, Gifu, 501-1194 Japan; 11 Brain Bank for Neurodevelopmental, Neurological and Psychiatric Disorders, United Graduate School of Child Development, Osaka University, Suita, Osaka 565-0871 Japan; 12 Department of General Internal Medicine, Okinawa Chubu Hospital, Uruma, Okinawa, 904-2293 Japan; 13 Laboratory of Structural Neuropathology, Tokyo Metropolitan Institute of Medical Science, Setagaya, Tokyo, 156-8506 Japan; 14 Department of Neurology, NHO Suzuka National Hospital, Suzuka, Mie 513-8501 Japan; 15 Department of Neurology, Faculty of Medicine and Graduate School of Medicine, Hokkaido University, Sapporo, Hokkaido 060-8638 Japan; 16 Institute for Chemical Reaction Design and Discovery (WPI-ICReDD), Hokkaido University, Sapporo, Hokkaido 001-0021 Japan; 17 Division of Neurology, Department of Brain and Neurosciences, Faculty of Medicine, Tottori University, Yonago, Tottori 683-8504 Japan; 18 Department of Neurology, NHO Osaka Toneyama Medical Center, Toyonaka, Osaka, 560-8552 Japan; 19 Department of Neurology, Tokyo Metropolitan Neurological Hospital, Fuchu, Tokyo, 183-0042 Japan; 20 Department of Neurology, NHO Sagamihara National Hospital, Sagamihara, Kanagawa 252-0392 Japan; 21 Department of Laboratory Medicine, National Center of Neurology and Psychiatry, National Center Hospital, Kodaira, Tokyo, 187-8551 Japan; 22 Department of Psychiatry, Kinoko Espoir Hospital, Kasaoka, Okayama, 714-0071 Japan; 23 Department of Neuropsychiatry, Okayama University Graduate School of Medicine, Dentistry and Pharmaceutical Sciences, Kita, Okayama, 700-8558 Japan; 24 Department of Neurology, NHO Akita National Hospital, Yurihonjo, Akita, 018-1393 Japan; 25 Laboratory of Neuropathology, Tokyo Metropolitan Institute of Medical Science, Setagaya, Tokyo, 156-8506 Japan; 26 Department of Neurology and Neurological Sciences, Tokyo Medical and Dental University, Bunkyo, Tokyo, 113-8519 Japan; 27 Department of Neurology, Oyamada Memorial Spa Hospital, Yokkaichi, Mie, 512-1111 Japan; 28 Department of Neurology, NHO Iou National Hospital, Kanazawa, Ishikawa 920-0192 Japan; 29 Department of Neurology, Ojiya Sakura Hospital, Ojiya, Niigata, 947-0041 Japan; 30 Department of Neurology, Brain Disease Center Agano Hospital, Agano, Niigata, 959-2221 Japan; 31 Department of Pathology, Brain Research Institute, Niigata University, Chuo, Niigata, 951-8585 Japan; 32 Department of Neurology, Clinical Neuroscience Branch, Brain Research Institute, Niigata University, Chuo, Niigata, 951-8585 Japan; 33 Department of Neurology, Tohoku University Graduate School of Medicine, Sendai, Miyagi 980-8574 Japan; 34 Department of Neurology, Fujita Health University School of Medicine, Toyoake, Aichi 470-1192 Japan; 35 Department of Clinical Research Education, Nagoya University Graduate School of Medicine, Nagoya, Aichi 466-8550 Japan; 36 Department of Neurology, Kariya Toyota General Hospital, Kariya, Aichi 448-8505 Japan; 37 Department of Neurology, Japanese Red Cross Aichi Medical Center Nagoya Daini Hospital, Nagoya, Aichi 466-8650 Japan; 38 Department of Neurology, Hekinan Municipal Hospital, Hekinan, Aichi 447-8502 Japan; 39 Department of Neurology, National Center for Geriatrics and Gerontology, Obu, Aichi 474-8511 Japan; 40 Department of Neurology, Graduate School of Medicine, The University of Tokyo, Bunkyo, Tokyo, 113-8655 Japan; 41 Department of Neurology, NHO Matsue Medical Center, Matsue, Shimane 690-8556 Japan; 42 Department of Neurology, NHO Higashinagoya National Hospital, Nagoya, Aichi 465-8620 Japan

**Keywords:** Corticobasal degeneration, Corticobasal syndrome, Magnetic resonance imaging, Asymmetry, Subcortical white matter hyperintensity

## Abstract

**Purpose:**

Due to the indistinguishable clinical features of corticobasal syndrome (CBS), the antemortem differentiation between corticobasal degeneration (CBD) and its mimics remains challenging. However, the utility of conventional magnetic resonance imaging (MRI) for the diagnosis of CBD has not been sufficiently evaluated. This study aimed to investigate the diagnostic performance of conventional MRI findings in differentiating pathologically confirmed CBD from its mimics.

**Methods:**

Semiquantitative visual rating scales were employed to assess the degree and distribution of atrophy and asymmetry on conventional T1-weighted and T2-weighted images. Additionally, subcortical white matter hyperintensity (SWMH) on fluid-attenuated inversion recovery images were visually evaluated.

**Results:**

In addition to 19 patients with CBD, 16 with CBD mimics (progressive supranuclear palsy (PSP): 9, Alzheimer’s disease (AD): 4, dementia with Lewy bodies (DLB): 1, frontotemporal lobar degeneration with TAR DNA-binding protein of 43 kDa(FTLD-TDP): 1, and globular glial tauopathy (GGT): 1) were investigated. Compared with the CBD group, the PSP-CBS subgroup showed severe midbrain atrophy without SWMH. The non-PSP-CBS subgroup, comprising patients with AD, DLB, FTLD-TDP, and GGT, showed severe temporal atrophy with widespread asymmetry, especially in the temporal lobes. In addition to over half of the patients with CBD, two with FTLD-TDP and GGT showed SWMH, respectively.

**Conclusion:**

This study elucidates the distinct structural changes between the CBD and its mimics based on visual rating scales. The evaluation of atrophic distribution and SWMH may serve as imaging biomarkers of conventional MRI for detecting background pathologies.

## Introduction

Corticobasal degeneration (CBD) is a neurodegenerative disorder characterized by the presence of cortical and striatal hyperphosphorylated 4-repeat (4R) tau deposition, especially astrocytic plaques and thread-like lesions, in the white matter and gray matter, accompanied by neuronal loss in the cortical regions and substantia nigra [1]. In contrast to the tuft-shaped astrocytes observed in progressive supranuclear palsy (PSP), astrocytic plaques are predominantly distributed in the prefrontal and premotor cortices in CBD [2].

As a member of the frontotemporal lobar degeneration (FTLD) spectrum, CBD exhibits diverse clinical phenotypes [3]. In addition to classical symptoms like ideomotor and limb-kinetic apraxia, alien limb, limb dystonia, myoclonus, and L-dopa unresponsive rigidity and bradykinesia (i.e., corticobasal syndrome [CBS]), patients with CBD may manifest various clinical manifestations including PSP syndrome, frontal behavioral-spatial syndrome, non-fluent/agrammatic variant of primary progressive aphasia, posterior cortical atrophy syndrome, and Alzheimer’s-like dementia [4, 5]. Adding to the complexity, other neurodegenerative disorders such as PSP, Alzheimer’s disease (AD), TAR DNA-binding protein of 43 kDa (TDP-43) proteinopathy, and dementia with Lewy bodies (DLB) can present with CBS indistinguishable from that observed in CBD [5–7]. Even with the introduction of new diagnostic criteria, accurately diagnosing CBD remains a challenge [4, 8, 9]. Consequently, the rate of correct antemortem diagnosis of CBD remains low, necessitating neuropathological examination for the accurate diagnosis of this condition.

Considering the diagnostic challenges based on clinical manifestations, the identification of objective biomarkers to differentiate these disorders becomes imperative. For the diagnosis of AD, positron emission tomography (PET) is utilized to detect amyloid- and tau-related pathologies [10, 11]. The detection of amyloid positivity is useful in distinguishing CBS caused by AD pathologies from CBS caused by non-AD pathologies [12]. Cardiac ^123^I-MIBG scintigraphy is a valuable method for assessing sympathetic denervation in patients with Lewy body disease [13, 14]. However, these methods are expensive and invasive owing to the associated radiation exposure. Furthermore, unlike AD and DLB, useful biomarkers for CBD, PSP, and TDP-43 proteinopathy have not yet been established. Compared with PET and scintigraphy, magnetic resonance imaging (MRI) serves as a readily accessible and noninvasive biomarker in routine clinical practice. MRI aids in the differentiation of neurodegenerative disorders by identifying the characteristic signal changes and atrophic patterns [15]. Therefore, investigating the utility of conventional MRI in discriminating between patients with CBD and those with CBS due to non-CBD pathologies (CBD mimics) is a relevant and important research endeavor. This retrospective study aimed to evaluate the diagnostic accuracy of MRI findings routinely used in clinical practice in patients with pathologically confirmed CBD and CBD mimics.

## Materials and methods

This study was conducted as a part of the Japanese validation study of the consensus criteria for CBD diagnosis (J-VAC study) within the framework of the Research Committee of CNS Degenerative Diseases and Research on Policy Planning and Evaluation for Rare and Intractable Diseases, Health, Labour, and Welfare Sciences Research Grants, the Ministry of Health, Labour and Welfare, Japan. A majority of the participating institutions in the J-VAC study were specialized in managing movement disorders, with the clinical diagnosis made by neurology specialists. Informed consent was obtained from the patients or bereaved families as an opt-out option on the website. The study was approved by the Ethics Committee of the National Hospital Organization Higashinagoya National Hospital (#27–8) and each institute, and was conducted in strict compliance with the ethical standards of the 1964 Declaration of Helsinki and its later amendments.

### Study population

The J-VAC study included 32 patients with CBD pathologies and 32 patients with CBS due to non-CBD pathologies (CBD mimics) from 48 institutions between 1996 and 2018 [16]. Patients with CBD mimics met the diagnostic criteria for CBS defined by the Mayo Clinic or the Cambridge [6, 17]. From this cohort, participants who underwent MRI, which included at least T1-weighted image (T1WI) and T2-weighted image (T2WI) scans, were recruited. Patients with insufficient MRI data (e.g., lack of whole-brain coverage) and with destructive lesions (e.g., cerebrovascular disorders and neoplasms), which could cause significant cerebral atrophy and signal changes on MRI scans, were excluded. As it was difficult to evaluate the distribution of atrophic changes, participants who underwent MRI at the advanced stage only were also excluded.

### Neuropathological analysis of CBD and CBD mimics

Formalin-fixed, paraffin-embedded glass slide specimens that were subjected to hematoxylin–eosin staining, Klüver-Barrera staining, Gallyas-Braak (G-B) silver staining, phosphorylated tau (AT8) staining, and amyloid β protein immunohistochemistry were reviewed by a group of well-experienced neuropathologists as previously described [16]. The neuropathological diagnoses of CBD, PSP, FTLD-TDP, AD, and DLB were made according to the established criteria [1, 18–21].

### MRI protocol and visual analyses

Owing to the retrospective nature of this study involving data from various institutions, the MRI protocols employed were not standardized. As a result, the parameters used in the study such as the magnetic field strength, repetition time, echo time, slice thickness, and field of view varied across the database. Therefore, the basic sequences, including T1WI, T2WI, and fluid-attenuated inversion recovery (FLAIR) images, were visually evaluated using the semiquantitative visual rating scales outlined below.

The four-point global cortical atrophy (GCA) scale was utilized to evaluate the degree of atrophy in the frontal, temporal, parietal, and occipital lobes [22]. The score was determined by visually evaluating the axial T1WIs. To assess the degree of atrophy in the hippocampus, the five-point Scheltens’ medial temporal atrophy (MTA) scale was evaluated mainly on coronal T1WIs [23]. Using these methods, assessments were conducted separately for each hemisphere, with the final score being the sum of bilateral hemispheres.

**Fig. 1:**
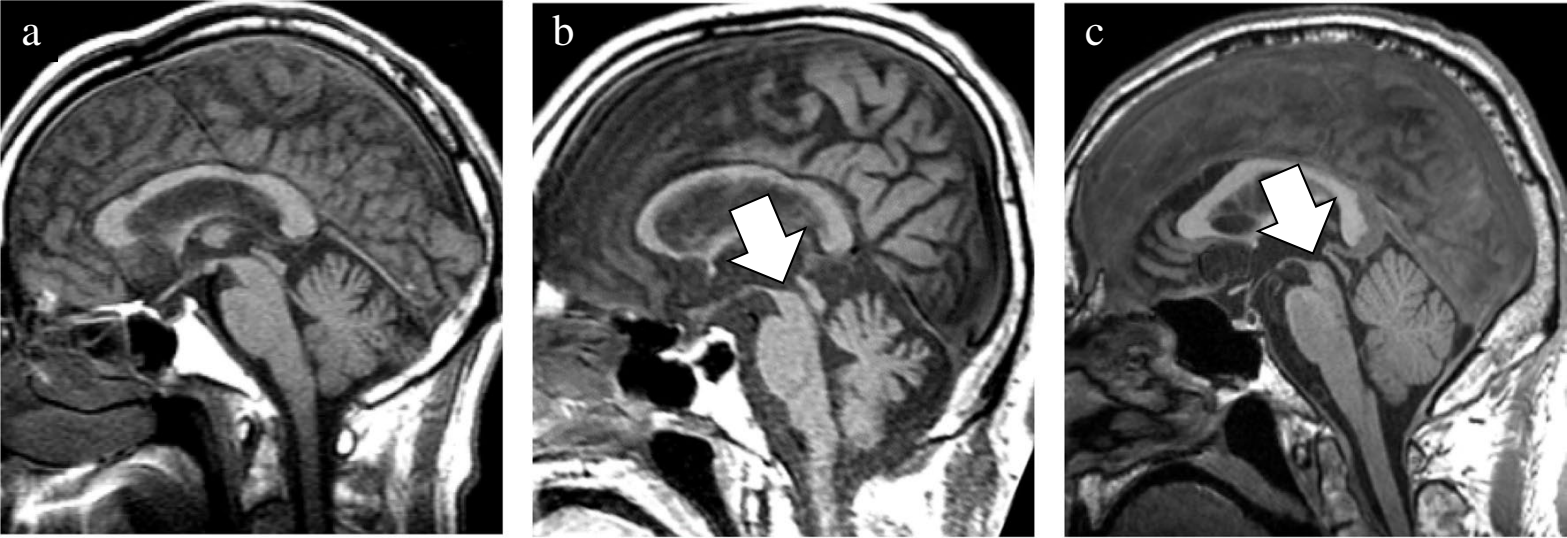
Visual rating scale of midbrain atrophy. The degree of midbrain atrophy was assessed using the three-point scale (0 = normal, 1 = mild, and 2 = severe). Compared with the normal midbrain in a patient with pathologically confirmed AD (**a**), mild midbrain atrophy showing a slight decrease in anteroposterior and/or superoinferior diameters was observed in a patient with pathologically confirmed CBD (**b**). By contrast, a definite decrease in these diameters was classified as severe midbrain atrophy in a patient with pathologically confirmed PSP (**c**). AD, Alzheimer’s disease; CBD, corticobasal degeneration; PSP, progressive supranuclear palsy

**Fig. 2:**
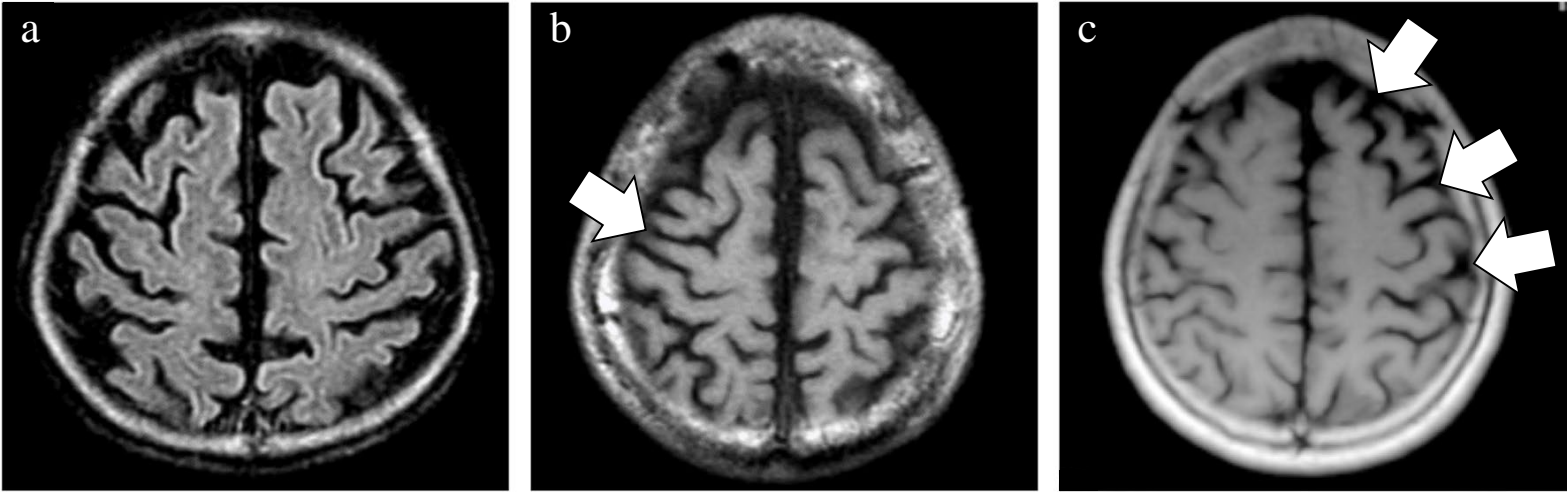
Visual rating scale of asymmetric atrophy. The degree of asymmetry in the frontal, temporal, parietal, and occipital lobes, and cerebral peduncle was assessed using a three-point scale (0 = normal, 1 = mild, and 2 = severe). In contrast to no obvious asymmetry in a patient with pathologically confirmed DLB (**a**), mild asymmetry and severe asymmetry were observed in patients with pathologically confirmed PSP (**b**) and CBD (**c**). CBD, corticobasal degeneration; DLB, dementia with Lewy bodies; PSP, progressive supranuclear palsy

In addition to the well-established GCA and MTA scales, newly devised rating scales were utilized to evaluate the presence of midbrain atrophy and asymmetric atrophy. The degree of midbrain atrophy was assessed using a three-point scale (0 = normal, 1 = mild, and 2 = severe) primarily on sagittal T1WIs (Fig. 1). Mild midbrain atrophy indicated a slight reduction in the anteroposterior and/or superoinferior diameters. By contrast, a definitive decrease in these diameters was classified as severe midbrain atrophy. The degree of asymmetry in the frontal, temporal, parietal, and occipital lobes, along with the cerebral peduncle, was assessed using a three-point scale (0 = normal, 1 = mild, and 2 = severe) on axial T1WIs (Fig. 2). Severe asymmetry indicated the definite dilation of the sulcus and/or narrowing of the gyrus between the two hemispheres. Conversely, a case with unremarkable asymmetry was rated as mild.

**Fig. 3:**
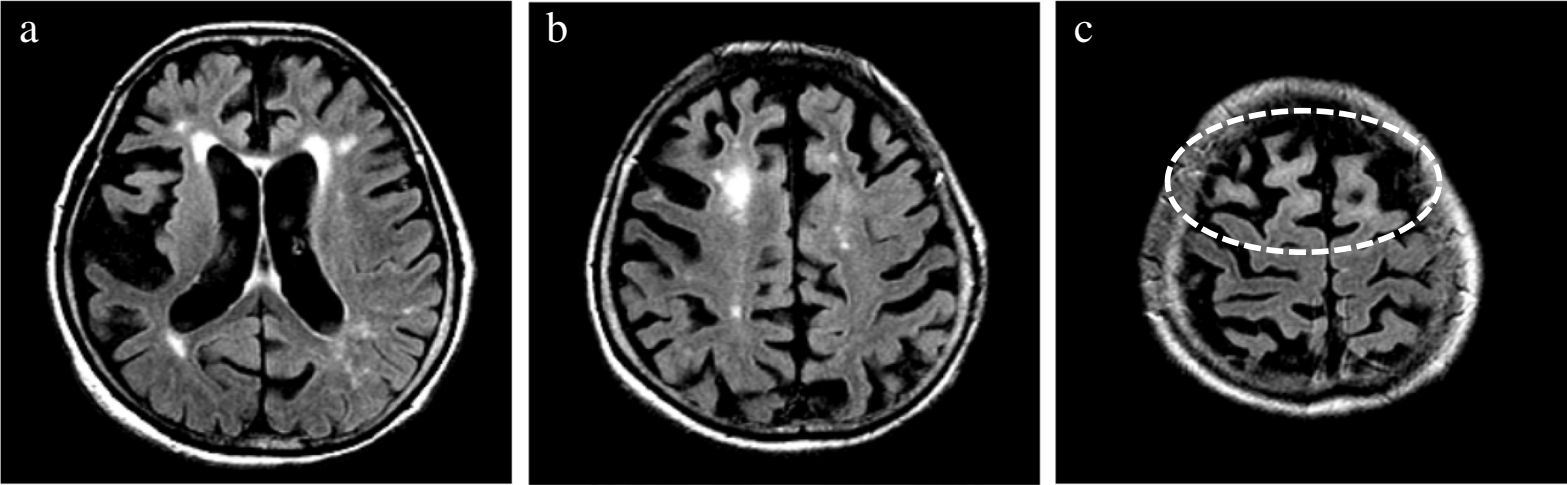
Evaluation of SWMH. The presence/absence of hyperintensity confined to the subcortical white matter (i.e., SWMH) was evaluated on an axial FLAIR image. In contrast to other lobes at lower convexity level (**a**, **b**), distinct SWMH of the bilateral frontal lobes at high convexity (**c**) was observed in a patient with pathologically confirmed CBD. CBD, corticobasal degeneration; SWMH, subcortical white matter hyperintensity

In addition, the presence/absence of subcortical white matter hyperintensity (SWMH) was visually evaluated on axial FLAIR images [24, 25]. Considering that hyperintensity in the periventricular and deep white matter can be attributed to aging and cerebral small vessel disease (i.e., leukoaraiosis), signal changes confined to the subcortical white matter were exclusively evaluated (Fig. 3).

The aforementioned visual rating scores were independently evaluated by two raters, a neuroradiologist and a neurologist, with 20 years (rater 1, K.S.) and 39 years (rater 2, AM.T.) of experience in neurodegenerative disorder neuroradiology, respectively. In cases of interobserver disagreement, final decisions were reached by unanimous consensus. Except for the research purpose, these two raters were blinded to the clinical and pathological diagnoses.

### Statistical analysis

Statistical analyses were performed using SPSS software (version 24.0; IBM Corp., Armonk, NY, USA). Student’s t-test was employed to compare normally distributed data, while the Mann–Whitney U test and Kruskal–Wallis test were employed to compare non-normally distributed data. Fisher’s exact test was used for nominal variables. When multiple comparisons showed a significant difference, an unpaired t-test or Mann–Whitney U test was also performed. The resulting *p* values were corrected using the Bonferroni method, and a *p* value of < 0.05 was considered significant.

## Results

### Participants’ data

**Fig. 4:**
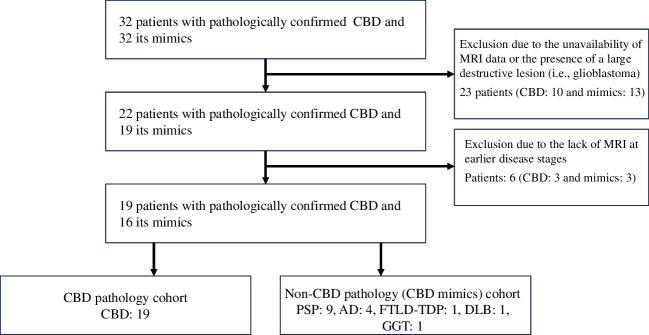
Flow chart showing the inclusion and exclusion of patients with pathologically confirmed CBD and those with mimics. AD, Alzheimer’s disease; CBD, corticobasal degeneration; DLB, dementia with Lewy bodies; FTLD-TDP, frontotemporal lobar degeneration with TAR DNA-binding protein of 43 kDa; GGT, globular glial tauopathy; PSP, progressive supranuclear palsy

First, 10 patients with CBD and 12 patients with CBD mimics were excluded from the J-VAC study cohort due to the unavailability of MRI data and the presence of large destructive lesions. Additionally, one patient with CBD mimics comorbid with glioblastoma was excluded. Second, three patients with CBD and three patients with CBD mimics were also excluded as only MRI data at the advanced stage were available. Ultimately, the study included a total of 19 patients with CBD and 16 patients with CBD mimics (Fig. 4).

Table 1 displays the pertinent clinicopathological characteristics of all participants. The antemortem clinical diagnosis of the CBD group predominantly included CBD or PSP. Similarly, CBD and CBS were the most prevalent clinical diagnoses in the CBD mimics group. The postmortem pathological diagnoses of the CBD mimics group comprised PSP (9 patients), AD (4 patients), FTLD-TDP (1 patient), DLB (1 patient), and globular glial tauopathy (GGT, 1 patient). Owing to the retrospective nature of the study, quantifying the participants’ neurological findings using advanced scales such as the unified Parkinson’s disease rating scale part III was challenging. Alternatively, the ratio of independent walking at the time of MRI scan was compared between the CBD group and CBD mimics group. No significant differences were observed in sex, age at MRI scan, disease duration at MRI scan, ratio of independent walking at MRI scan, or the time from the MRI scan until death between the CBD group and CBD mimics group.

**Table 1:** Participants’ characteristics

	CBD pathology	CBS with non-CBD pathology (CBD mimics)	*p* value
Number	19	16	N.A
Male/Female	9/10	13/3	0.08^a^
Diagnosis	Clinical diagnosis	Main neuropathology	N.A
	CBD/CBS 8	PSP 9	
	PSP 7	AD 4	
	FTD 2	FTLD-TDP 1	
	AD 1	DLB 1	
	CVD 1	GGT 1	
Age at MRI scan (y)	69.2 ± 6.9	73.2 ± 8.3	0.13^b^
Disease duration at MRI scan (y)	2.4 ± 2.0	3.6 ± 2.3	0.13^b^
Ratio of independent walking at	43%(6/14)	50%(6/12)	0.51^a^
MRI scan^d^			
Time from MRI scan until death (y)	5.0 (2.3, 5.8)	3.0 (2.0, 6.0)	0.57^c^

Data are expressed as absolute number, mean ± standard deviation, or median (1st quartiles and 3rd quartiles)

*AD* Alzheimer’s disease, *CBD* corticobasal degeneration, *CBS* corticobasal syndrome, *CVD* cerebrovascular disorder, *DLB* dementia with Lewy bodies, *FTD* frontotemporal dementia, *FTLD* frontotemporal lobar degeneration, *GGT* globular glial tauopathy, *MRI* magnetic resonance imaging, *N.A.* not applicable, *PSP* progressive supranuclear palsy, *TDP-43* TAR DNA-binding protein-43, *y* year

^a^Fisher’s exact test; ^b^Student’s t-test; ^c^Mann-Whitney U test; ^d^Clinical course was not available in five patients with CBD and four patients with CBD mimics

### MRI analysis data

**Fig. 5:**
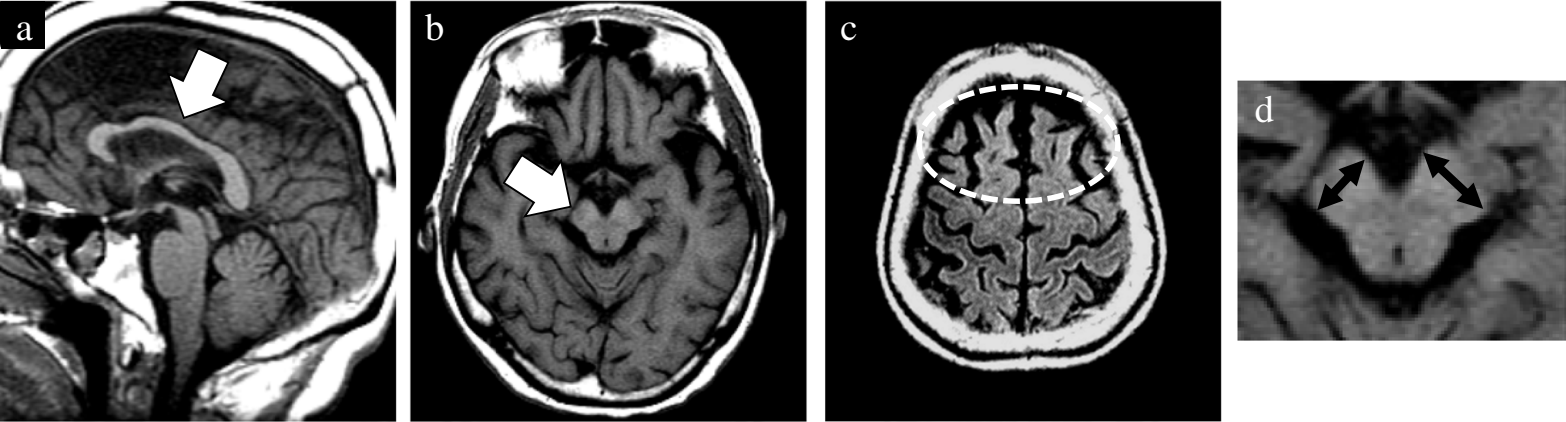
A representative case of radiologically typical CBD. This 69-year-old female patient obtained an antemortem clinical diagnosis of AD. After the postmortem pathological examination, it was proved that main pathology of this patient was CBD. MRI was performed one year after the symptom onset. In contrast to mild atrophy of the callosal body (arrow, **a**), sagittal T1WI showed relatively preserved midbrain. Axial T1WI and FLAIR image showed right dominant asymmetric atrophy of the cerebral peduncle and frontal lobe (arrow, **b**). Additionally, mild SWMH was detected in the frontal lobe at high convexity (circle, **c**). A magnified view was exhibited to emphasize the asymmetry of the short axis in the cerebral peduncles (double sided arrows, **d**). a, sagittal T1WI; b, axial T1WI; c, axial FLAIR image; AD, Alzheimer’s disease; CBD, corticobasal degeneration; FLAIR, fluid-attenuated inversion recovery; SWMH, subcortical white matter hyperintensity; T1WI, T1-weighted image

**Fig. 6:**
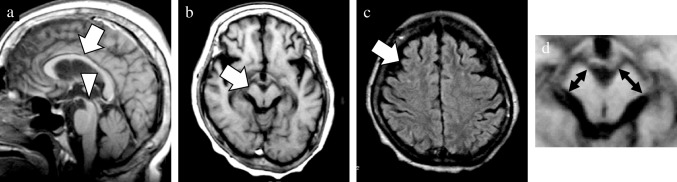
A representative case of radiologically atypical CBD. This 67-year-old female patient obtained an antemortem diagnosis of CBD. MRI was performed four years after the symptom onset. In addition to atrophy of the callosal body (arrow, **a**), mild midbrain atrophy was also detected on sagittal T1WI (arrowhead, **b**). In contrast to right dominant asymmetric atrophy of the cerebral peduncle and frontal lobe (arrows, **b**, **c**), no obvious SWMH was noted on axial FLAIR image (**c**). A magnified view was exhibited to emphasize the asymmetry of the short axis in the cerebral peduncles (double sided arrows, **d**). **a**, sagittal T1WI; **b**, axial T1WI; **c**, axial FLAIR image; CBD, corticobasal degeneration; FLAIR, fluid attenuated inversion recovery; SWMH, subcortical white matter hyperintensity; T1WI, T1-weighted image

**Fig. 7:**
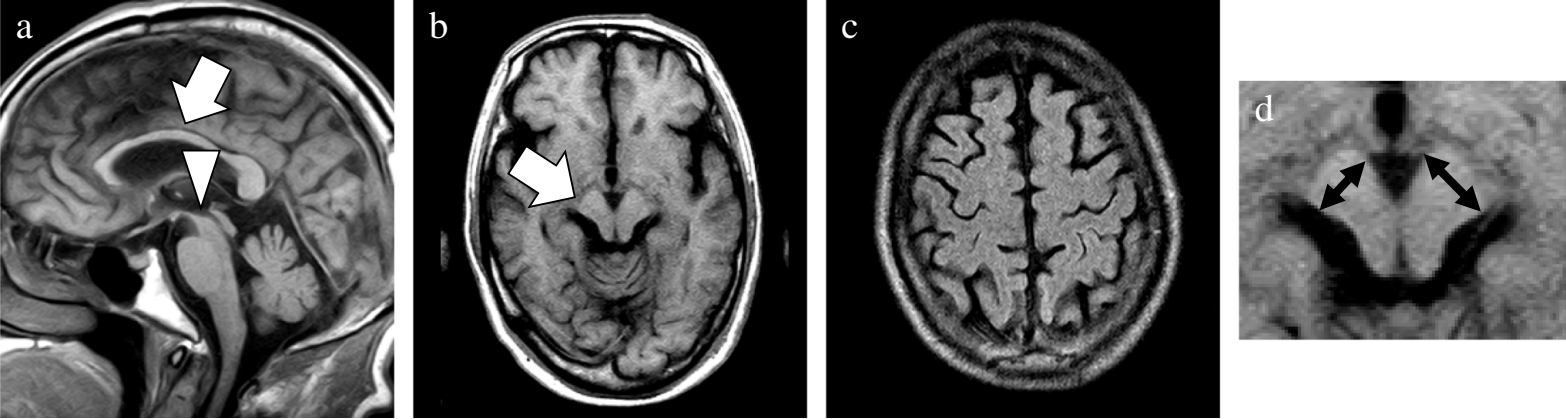
A representative case of CBD mimics. This 71-year-old male patient obtained a postmortem diagnosis of PSP. MRI was performed two years after the symptom onset. Sagittal and axial T1WI (**a**, **b**), and axial FLAIR image (**c**) showed atrophy of the midbrain and callosal body (arrow and arrowhead, **a**), and right dominant asymmetric atrophy of the cerebral peduncle without obvious SWMH (arrow, **b**). These MRI findings were very similar to those of the patient described in Fig. 6. The case of this patient was exhibited to clarify the challenges when differentiating CBD without SWMH from its mimics, especially PSP. A magnified view was exhibited to emphasize the asymmetry of the short axis in the cerebral peduncles (double sided arrows, **d**). **a**, sagittal T1WI; **b**, axial T1WI; **c**, axial FLAIR image; CBD, corticobasal degeneration; FLAIR, fluid attenuated inversion recovery; PSP, progressive supranuclear palsy; SWMH, subcortical white matter hyperintensity; T1WI, T1-weighted image

**Fig. 8:**
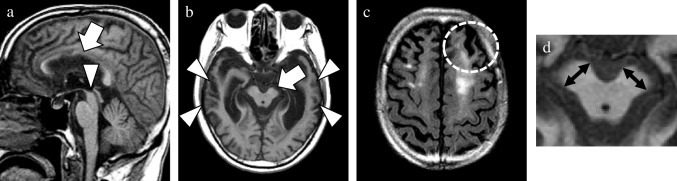
A representative case of CBD mimics. This 74-year-old male patient obtained a postmortem diagnosis of FTLD-TDP. MRI was performed three years after the symptom onset. The confusable atrophy of the midbrain and callosal body (arrow and arrowhead, **a**), asymmetric atrophy of the left cerebral peduncle (arrow, **b**) and frontal lobe, and SWMH in the left frontal lobe at high convexity (circle, **c**) were detected. The severity of bilateral temporal lobe atrophy (arrowheads, **b**) significantly differed from that of CBD. This patient was exhibited to clarify the different atrophy patterns in the CBD mimics group, especially the non-PSP subgroup. A magnified view was exhibited to emphasize the asymmetry of the short axis in the cerebral peduncles (double sided arrows, **d**). **a**, sagittal T1WI; **b**, axial T1WI; **c**, axial FLAIR image; CBD, corticobasal degeneration; FLAIR, fluid attenuated inversion recovery; FTLD-TDP, frontotemporal lobar degeneration with TAR DNA-binding protein of 43 kDa; SWMH, subcortical white matter hyperintensity; T1WI, T1-weighted image

As the main neuropathology of more than half of the patients in the CBD mimics group was PSP, this group was further categorized into PSP-CBS and non-PSP-CBS subgroups. The MRI findings in the CBD and two CBD mimics subgroups are shown in Table 2. Compared with the PSP-CBS subgroup, the CBD group tended to show a higher SWMH ratio (56% vs 0%; *p* = 0.060). In the CBD group, SWMH was exclusively distributed in the frontal or parietal lobe at high convexity. Its sensitivity, specificity and accuracy to diagnose CBD were 56%, 86%, and 69%, respectively. Compared with the non-PSP-CBS subgroup, the CBD subgroup displayed relatively lower temporal GCA score and degree of asymmetry in the temporal and occipital lobes (0.5 [0.3–1.0] versus 1.5 [1.0–2.3], 0.0 [0.0–0.5] versus 1.0 [0.0–1.5], and 0.0 [0.0–0.0] versus 0.0 [0.0–0.0]; *p* = 0.073, *p* = 0.073 and *p* = 0.082, respectively). Confusable SWMH was detected in one patient with FTLD-TDP and one with GGT. Among the CBD mimics subgroups, the non-PSP-CBS subgroup showed higher frontal, temporal, and occipital lobe GCA scores (1.5 [1.0–2.5] versus 0.5 [0.5–1.0], 1.5 [1.0–2.3] versus 0.0 [0.0–0.5], and 0.5 [0.0–1.0] versus 0.0 [0.0–0.0]; *p* = 0.043, *p* = 0.011, and *p* = 0.019, respectively). By contrast, the degree of midbrain atrophy was most severe in the PSP-CBS subgroup (2.0 [1.0–2.0] versus 0.0 [0.0–0.3], and 2.0 [1.0–2.0] versus 0.0 [0.0–0.0]; *p* = 0.016 and *p* = 0.014, respectively). The (weighted) kappa of the above-mentioned visual analyses for the interrater reliability of the two raters was high, ranging from 0.77 to 1.00 in this study. The representative MRI findings of patients with pathologically confirmed CBD and CBD mimics are presented in Figs. 5, 6, 7 and 8.

**Table 2:** MRI findings

	CBD pathology	CBS with non-CBD pathology (CBD mimics)		
		PSP-CBS	non-PSP-CBS	*p* value
GCA scale				
Frontal lobe	1.0 (1.0–1.5)	0.5 (0.5–1.0)	1.5 (1.0–2.5)^f^	0.037^a^
Temporal lobe	0.5 (0.3–1.0)	0.0 (0.0–0.5)	1.5 (1.0–2.3)^g,h^	0.013^a^
Parietal lobe	1.0 (0.5–1.3)	1.0 (0.5–1.0)	1.5 (1.3–2.5)	0.11^a^
Occipital lobe	0.0 (0.0–0.0)	0.0 (0.0–0.0)	0.5 (0.0–1.0)^i^	0.020^a^
MTA scale^c^	1.0 (0.2–2.4)	1.5 (1.4–2.5)	1.5(1.0–1.8)	0.17^a^
Midbrain atrophy scale^d^	0.0 (0.0–0.3)	2.0 (1.0–2.0)^j,k^	0.0 (0.0–0.0)	0.006^a^
Asymmetry scale				
Frontal lobe	1.0 (1.0–1.0)	2.0 (1.0–2.0)	1.0 (1.0–2.0)	0.35^a^
Temporal lobe	0.0 (0.0–0.5)	0.0 (0.0–1.0)	1.0 (0.0–1.5)^l,m^	0.003^a^
Parietal lobe	1.0 (0.0–2.0)	0.0 (0.0–2.0)	2.0 (1.0–2.0)^n^	0.007^a^
Occipital lobe	0.0 (0.0–0.0)	0.0 (0.0–0.0)	0.0 (0.0–0.0)^o,p^	0.002^a^
Cerebral peduncle	1.0 (0.0–2.0)	2.0 (1.0–2.0)	1.0 (0.0–1.5)	0.27^a^
SWMH^e^	10/18 (56%)^q^	0/7 (0%)	2/7 (29%)	0.033^b^

Data are expressed as the absolute number or median (1st quartiles and 3rd quartiles)

Imaging findings showing statistical significance are highlighted in gray color

*CBD* corticobasal degeneration, *CBS* corticobasal syndrome, *GCA* global cortical atrophy, *MRI* magnetic resonance imaging, *MTA* medial temporal atrophy, *PSP* progressive supranuclear palsy, *SWMH* subcortical white matter hyperintensity

^a^Kruskal-Wallis test; ^b^Fisher’s exact test; ^c^MTA scale was not applicable in nine patients with CBD and one patient with CBD mimics; ^d^Midbrain atrophy was not applicable in three patients with CBD; ^e^SWMH was not applicable in one patient with CBD and two patients with CBD mimics; ^f^*p* = 0.043 PSP-CBS subgroup versus non-PSP-CBS; ^g^*p* = 0.073 CBD versus non-PSP-CBS; ^h^*p* = 0.011 PSP-CBS versus non-PSP-CBS; ^i^*p* = 0.019 PSP-CBS versus non-PSP-CBS; ^j^*p* = 0.016 CBD versus PSP-CBS; ^k^*p* = 0.014 PSP-CBS versus non-PSP-CBS; ^l^*p* = 0.073 CBD versus non-PSP-CBS; ^m^*p* = 0.011 PSP-CBS versus non-PSP-CBS; ^n^*p* = 0.005 CBD versus non-PSP-CBS; ^o^*p* = 0.082 CBD versus non-PSP-CBS; ^p^*p* = 0.019 PSP-CBS versus non-PSP-CBS; ^q^*p* = 0.060 CBD versus PSP-CBS; f–p were evaluated by Mann–Whitney U test with Bonferroni correction; q was evaluated by Fisher’s exact test with Bonferroni correction

## Discussion

**Fig. 9:**
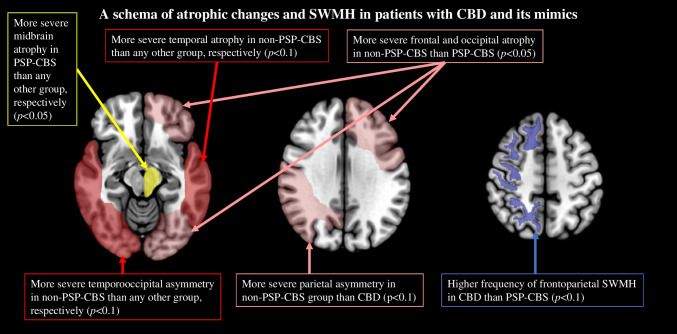
A schema of atrophic changes and SWMH in patients with CBD and its mimics. In this scheme, asymmetry and signal change were colored in the right hemisphere, and degree of brain atrophy was colored in the left hemisphere, respectively. CBD, corticobasal degeneration; CBS, corticobasal syndrome; PSP, progressive supranuclear palsy; SWMH, subcortical white matter hyperintensity

This study aimed to differentiate patients with pathologically confirmed CBD from those with CBD mimics using semiquantitative visual rating scales. Various rating scales reflecting the degree of atrophy and asymmetry were not useful for differentiating CBD and its mimics. Conversely, the abnormal subcortical white matter signal on axial FLAR (i.e., SWMH) was higher in patients with CBD than those with its mimics. Furthermore, severe midbrain in patients with the PSP-CBS subgroup, and severe temporal atrophy and widespread asymmetry in patients with the non-PSP-CBS subgroup were higher than those in patients with CBD. These results suggest that SWMH and particular atrophic distribution could be a valuable contributor to the differentiation between CBD and its mimics (Fig. 9).

CBD is a predominantly sporadic 4R tauopathy, clinically classified as one of the atypical parkinsonian syndromes. This condition is characterized by an asymmetrical presentation of akinetic-rigid syndrome, involuntary movements, and signs of cortical dysfunction [26]. However, this clinical feature is not specific to CBD and can be caused by various pathologic conditions, including PSP, AD, FTLD-TDP, and DLB [5–7]. Therefore, the term “CBS” instead of “CBD” was established to encompass the constellation of symptoms associated with this clinical phenotype. The lack of specific clinical symptoms and biomarkers contributes to the challenging antemortem diagnosis of CBD.

Asymmetry is a key imaging feature of CBD. Previous studies, evaluating pathologically confirmed patients, have elucidated the presence of asymmetric atrophy in patients with CBD [24, 25, 27]. Voxel-based morphometry (VBM) and volumetry are employed for the objective investigation of this asymmetric atrophy [28–30]. Additionally, distinctive signal changes in the white matter and subthalamic nuclei have been reported [25, 31]. These results underscore the efficacy of MRI in detecting characteristic structural changes associated with CBD.

However, like a clinical presentation, comparable imaging findings have been documented in both pathologically confirmed CBD and CBS due to non-CBD pathologies. Asymmetric atrophy, which is the most well-known finding of CBD, can be observed in patients with PSP, AD, and FTLD-TDP [30, 32–34]. Hence, the degree and distribution of atrophy were also evaluated in this study. Visual rating scales delineate distinct atrophic profiles among the CBD and CBD mimics subgroups. Compared with the CBD and PSP-CBS subgroups, the non-PSP-CBS subgroup showed more severe asymmetric atrophy, especially in the temporal lobe. This discrepancy aligns with the findings of a previous VBM study, demonstrating more extensive asymmetric temporal, parietal, and occipital atrophy in AD and FTLD-TDP than in CBD and PSP [28]. Actually, the non-PSP-CBS subgroup in this study comprised patients with AD and FTLD-TDP. The pathological changes of these disorders can frequently impair the temporal region. Consequently, severe asymmetric atrophy, particularly involving the temporal region, may become a key feature for distinguishing CBD from its non-PSP-CBS mimics.

Midbrain atrophy, a well-known imaging finding of PSP, has been reported in patients with pathologically confirmed CBD [25, 35]. In this study, the degree of midbrain atrophy is more severe in patients with PSP than in those with CBD. However, the degree of midbrain atrophy depends on the background pathologies and clinical phenotypes [36]. Patients with atypical PSP may exhibit mild midbrain atrophy indistinguishable from that of patients with CBD and vice versa.

Compared with the above-mentioned atrophic changes, changes in white matter signal have not garnered much attention as an imaging feature of CBD. However, some studies have reported white matter signal changes in patients with pathologically confirmed CBD [24, 25, 31]. Notably, just over half of the patients with CBD showed SWMH, and this ratio was relatively higher than that of patients with PSP-CBS in this study. Furthermore, consistent with the result of a previous study comparing patients with pathologically confirmed CBD and those with PSP, none of the patients with PSP showed SWMH [25]. SWMH was not also observed in patients with AD and DLB. In CBD brains, tau-positive fine threads extensively distribute in the cerebral cortices, subcortical white matter, basal ganglia, thalamus, and brainstem. These threads cause not only atrophic changes but also loss of myelin beneath the subcortical white matter including U-fibers. This subcortical white matter degeneration seems to cause MRI signal changes (i.e., SWMH) in patients with CBD. In contrast, such pathological changes are not generally observed in PSP and AD brains, which constitute the majority of CBD-mimics. Considering the pathogenesis of the signal change, this finding can be useful for the differentiation of CBD and other disorders. Thus, SWMH can serve as a clue for the diagnosis of CBD, but caution is warranted when interpreting this finding. First, considering the nonspecific signal changes (i.e., leukoaraiosis) observed in the periventricular and deep white matter, signal changes localized to the subcortical region should be evaluated. Second, patients with non-CBD pathologies may exhibit confusable subcortical white matter signal changes [34, 37]. In this study, two patients with CBD mimics (one who developed FTLD-TDP and another who developed GGT) were judged as having a positive SWMH. In diagnosing patients with asymmetric atrophy and SWMH, not only CBD but also FTLD-TDP and GGT should be considered as one of the differential diagnoses.

The application of semiquantitative visual rating scales in patients with pathologically confirmed cases represents a notable strength of this study. A previous study has already clarified the close correlation between the regional gray matter atrophy on VBM and visual assessment on visual rating scales [38]. Therefore, it is plausible to consider that visual assessment by experienced rates has diagnostic capabilities similar to VBM. Semiquantitative visual rating scales provide a clear delineation of the varying degrees and distributions of atrophy and asymmetry between the CBD and CBD mimics subgroups without the need for specialized software. Nonetheless, some limitations are associated with the retrospective nature of this study. First, the number of participants was relatively small, especially those with AD and FTLD-TDP. The visual rating scale scores demonstrated more pronounced atrophy and asymmetry of the temporal lobe in the non-PSP-CBS subgroup, aligning with the findings of the VBM study [28]. However, the non-PSP-CBS subgroup included those with nonuniform conditions, and the number of participants in this group was relatively small. A larger number of cases is necessary to accurately assess the atrophic and asymmetric findings in the non-PSP-CBS. Second, inconsistent MRI protocols made it impossible to perform more sophisticated analyses such as VBM and diffusion tensor imaging. It should be noted that even using semiquantitative visual rating scales, it is not easy to detect subtle imaging abnormalities for inexperienced raters. Considering the difficulty to detect the subtle SWMH and asymmetric atrophy by visual assessment, establishment of supplementary techniques such as VBM, volumetry, and deep learning to support visual assessment is a future challenge for the imaging diagnosis of CBD. The exploration of MRI parameters such as FLAIR to detect subtle SWMHs remains challenging. Therefore, a large multicenter prospective cohort study with a uniform MRI protocol is required to further investigate the imaging differences between the CBD and CBD mimics.

## Conclusion

This study clarifies the different structural changes between the CBD and its mimics based on visual rating scales. The evaluation of atrophic distribution and SWMH may serve as imaging key features in conventional MRI.

## Data Availability

The data used for MRI and pathological analyses are available from the authors and may be provided upon reasonable request to the corresponding author.
